# Using Heart Rate to Measure Stress in Healthcare Workers Wearing PAPRs and N95 Masks: Insights from a Randomized Trial

**DOI:** 10.3390/s26113531

**Published:** 2026-06-03

**Authors:** Rodrigo M. A. Almeida, Rafael Rocha Maciel, Carlos Henrique Valério Moraes, Caroline Lopes Ciofi-Silva, Naila A. Oliveira, Giulia M. Mainardi, Luciana Cordeiro, Anna Sara Shafferman Levin, Amy I. Price, Ying Ling Lin, Maria Clara Padoveze

**Affiliations:** 1 Institute of Systems Engineering and Information Technology, Federal University of Itajubá—UNIFEI, Itajubá 37500-903, MG, Brazil; rafaelrocha@unifei.edu.br (R.R.M.); valerio@unifei.edu.br (C.H.V.M.); 2 School of Nursing, University of Campinas—UNICAMP, Campinas 13083-887, SP, Brazil; clciofi@unicamp.br; 3 São Leopoldo Mandic Medical School, Araras 13600-001, SP, Brazil; nailaa.oliveira@gmail.com; 4 São Leopoldo Mandic Medical School, Limeira 13482-890, SP, Brazil; 5 School of Nursing, University of São Paulo—USP, São Paulo 05403-000, SP, Brazil; gmmainardi@gmail.com (G.M.M.); lucordeiro.to@gmail.com (L.C.); anna@usp.br (A.S.S.L.); padoveze@usp.br (M.C.P.); 6 Dartmouth Institute for Health Policy and Clinical Practice, Lebanon, NH 03766, USA; amy.i.price@dartmouth.edu; 7 World Health Organization—WHO, 1211 Geneva, Switzerland; liny@who.int

**Keywords:** wearable sensors, wearable health monitoring, personal protective equipment

## Abstract

This study investigates the impact of different types of personal protective equipment (PPE), specifically Powered Air-Purifying Respirators (PAPRs) and traditional N95 masks with face shields, on the physiological stress responses of healthcare workers (HWs) during the COVID-19 pandemic. Utilizing an interventional randomized crossover trial design, the research encompasses a simulation phase with ten participants followed by field testing involving thirty frontline healthcare professionals in a tertiary-care hospital setting. Heart rate (HR) and movement data were collected through smartwatches, while trained observers recorded the duration and nature of various activities undertaken during simulations. Data analysis employed statistical techniques, including Principal Component Analysis (PCA) and t-Distributed Stochastic Neighbor Embedding (t-SNE), to explore potential correlations between PPE type, HR, and movement. Clustering validation measures such as the Calinski–Harabasz, Davies–Bouldin, and Silhouette scores were applied to evaluate the difference between each type of PPE. The results indicated no significant differentiation in HR responses between the two PPE types. However, because HR may lack the sensitivity to fully capture variations in cognitive load or stress, these findings should be interpreted as an exploratory baseline. Additionally, no clear distinctions were observed regarding individual user responses or the activities performed, even when considering movement data. Although the findings imply non-inferiority of the examined PPE, future research including heart rate variability as a more comprehensive indicator of stress would be informative. This research contributes valuable insights into PPE selection and its implications for healthcare worker performance and well-being in high-stress environments, ultimately aiming to inform guidelines and training programs to enhance healthcare delivery during infectious disease outbreaks.

## 1. Introduction

The ongoing COVID-19 pandemic underscores the critical importance of effective personal protective equipment (PPE) for healthcare workers (HCWs) on the frontlines. Among the various options available, Powered Air-Purifying Respirators (PAPRs) and traditional PPE, such as the N95 in combination with face shields, have been widely utilized. The selection of appropriate PPE is essential for protecting healthcare professionals from viral exposure. However, we also need to measure the potential physiological and psychological impacts associated with prolonged use. In this context, understanding the effects of different PPE types on stress levels and cognitive load becomes paramount, particularly as these factors can influence performance and decision-making in high-stress environments. Measuring heart rate variability over time while wearing PPE may reveal an adaptive response indicative of the learning curve required to adapt to equipment in high-stress, resource-limited environments.

This study employs an interventional randomized crossover trial to assess the impact of different PPE types on healthcare workers by measuring their heart rate (HR) as a biomarker of stress. The trial consists of two phases: a simulation study involving ten participants and field testing with thirty frontline healthcare workers in a tertiary-care hospital setting. During the simulations, HR and movement data were collected using smartwatches, while trained observers documented the duration and nature of various activities undertaken by participants.

To analyze the data collected, techniques such as Principal Component Analysis (PCA) and t-Distributed Stochastic Neighbor Embedding (t-SNE) were employed. These analytical methods aim to identify correlations and classifications within the collected data. Furthermore, clustering validation measures will be applied to ensure the robustness of the findings, which will assist in understanding the significance of the results in the context of PPE usage and its implications for healthcare workers’ well-being.

Ultimately, this research seeks to contribute to the existing literature on PPE by exploring not only the physiological implications of different PPE types but also the broader ramifications for healthcare professionals operating in high-pressure environments. By shedding light on these critical issues, the study aims to inform future PPE guidelines and training programs, ensuring that healthcare workers are equipped with both the protective gear and the knowledge necessary to navigate the challenges posed by infectious disease outbreaks.

### 1.1. Literature: HR as Stress Indicator

The use of heart rate (HR) and heart rate variability (HRV) as indicators of stress has been long studied, with one study [1] claiming, “When heart rate is used in combination with the recording of job activities, changes in heart rate can indicate reactions to psychological stressors”. Other researches focus on both short-term fluctuations over periods of just a few seconds [2], and comparisons with daily baseline patterns [3]. The physiological connection between brain function and cardiac activity was reinforced by a meta-analysis conducted in 2011, which emphasized the importance of heart rate variability (HRV) as a potential biomarker for stress [4]. Since then, this correlation has been further substantiated, solidifying HRV’s role as a reliable marker for stress over regular HR.

Several studies have employed HRV to assess stress in various contexts, such as in drivers [5], athletes [6], airline pilots [7], and students [8,9], demonstrating its effectiveness across diverse scenarios. These studies highlight HRV’s capacity to reflect physiological responses to cognitive and physical stressors.

More recently, there has been significant progress in detecting stress using machine learning methods [10]. Research has utilized data collected from wearable devices to develop models capable of identifying stress patterns [11,12,13]. The highest-performing models to date have employed advanced classifiers such as deep learning Long Short-Term Memory (LSTM) networks [12], Support Vector Machines (SVMs) [11], and Random Forests [13]. However, given that these approaches rely on supervised learning, which requires labeled datasets and manual feature extraction, there is growing interest in unsupervised machine learning techniques. These unsupervised methods have shown promising results, achieving performance levels comparable to supervised approaches in stress detection [14].

Building on these foundations, recent research has prioritized multimodal sensor fusion, integrating signals such as Electrodermal Activity (EDA), heart rate variability (HRV), and skin temperature to achieve higher diagnostic accuracy than single-metric approaches [15]. Furthermore, contemporary studies emphasize ecological validity by transitioning from laboratory simulations to monitoring individuals in free-living environments utilizing machine learning for stress classification [16]. Specifically, commercial smartwatches have demonstrated utility in tracking physiological burden and heat-related stress for individuals wearing personal protective equipment (PPE) under practical testing conditions [17]. However, since complex multi-sensor setups can be difficult to scale in active clinical settings, it is crucial to determine if accessible, single-metric HR data can independently identify the physiological impacts of different respiratory protections.

### 1.2. PPE Pandemic Increase

Personal protective equipment (PPE) is broadly defined by the World Health Organization (WHO) as “any equipment (for example, gloves) worn by a person to protect that individual from exposure to one or more hazards” [18]. In healthcare settings, the proper and effective usage of PPE is influenced by a range of factors, including availability, user compliance, and the specific hazards encountered [19].

During the COVID-19 pandemic, the reliance on PPE by healthcare workers (HCWs) increased significantly due to the heightened risk of viral transmission. The frequency and perceived importance of PPE utilization grew as a necessary measure to mitigate exposure risks [20], with HCWs recognizing its critical role in ensuring safety [21]. This heightened awareness was reflected in the decrease in instances of non-use of PPE between the first and second waves of the pandemic, as the importance of consistent PPE usage became more evident [22].

However, the prolonged and intense use of PPE, particularly masks, has been associated with several adverse effects. These effects include skin injuries, respiratory discomfort (dyspnea), chest tightness, and frequent headaches [23]. Specifically, studies have identified a notable correlation between the extended use of PPE and the occurrence of headaches among healthcare professionals [24,25]. Given these challenges, there is a pressing need to enhance the comfort and tolerability of PPE while maintaining its protective functions [26].

Recent studies demonstrate the effectiveness of heart rate variability (HRV) training in reducing stress and improving resilience among healthcare professionals. A pilot study in January 2025 found that a Mindfulness-Oriented Professional Resilience program incorporating HRV biofeedback principles significantly improved the professional quality of life, including increased compassion, satisfaction, and reduced burnout among veterans recovering from PTSD [27]. Additionally, research from December 2024 showed that a brief HRV biofeedback intervention delivered via telemedicine was effective in reducing anxiety, depression, and stress among frontline healthcare workers during the COVID-19 pandemic [28].

## 2. Methodology

The study was submitted and approved by the ethical research committees of the World Health Organization (WHO) Ethics Review Committee for COVID-19 (Approval #0100). The multisite data management and security plan was agreed upon by the WHO, individual institutional IRBs, and Withings. Identifiable data was replaced with numerically coded identifiers.

This study employed the Withings Steel HR^®^ smartwatch [29] to continuously monitor heart rate (measured in beats per minute, BPM) and physical activity (measured in steps) of participants. Due to the unavailability of heart rate variability (HRV) measurements from the smartwatch, we focused solely on regular heart rate (HR) data for this analysis. Consequently, this is an exploratory study, limited by the HR’s sensitivity to detect subtle stress differences between PPE types. The smartwatch collected heart rate data during simulated healthcare tasks and for at least one full day following the simulation to establish a baseline for comparison.

The experimental design utilized a controlled simulation scenario in which each participant completed two separate runs, with the sole variable being the type of PPE worn. In one run, participants donned a Powered Air-Purifying Respirator (PAPR), while in the other, they wore an N95 respirator and face shield. To minimize bias related to learning effects from repeated tasks, participants were randomly assigned to start with either the PAPR or the N95 respirator and face shield. No significant differences were observed in the execution of activities between the first and second rounds, suggesting that potential adaptation effects were effectively mitigated by the randomization strategy. Consequently, this crossover design ensured that any observed differences in stress, as indicated by heart rate, could be attributed to the type of PPE used rather than to familiarity with the tasks.

**Figure 1:**
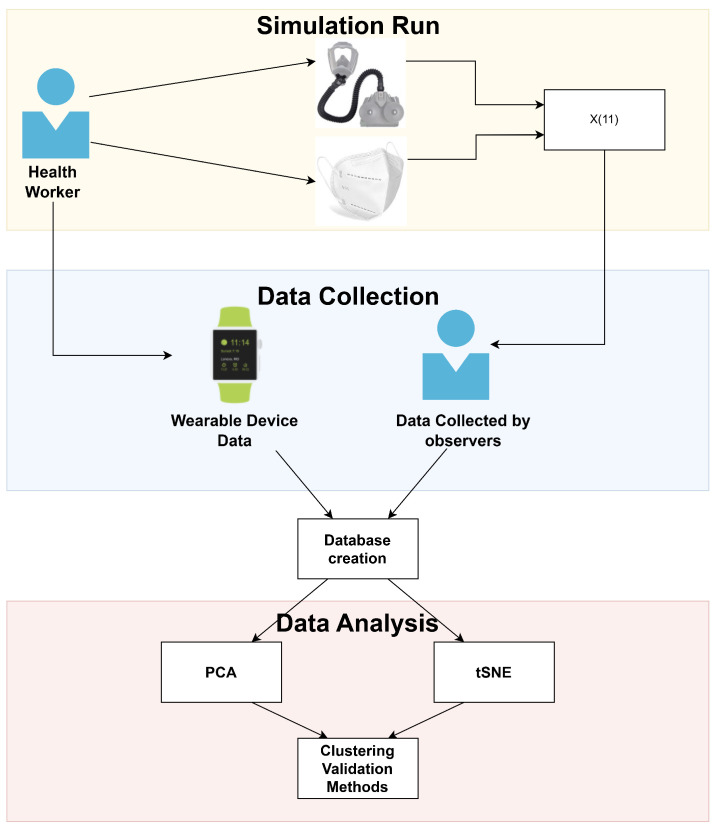
Methodology flowchart.

Heart rate measurements were continuously recorded throughout the simulation using the smartwatch. The primary objective was to assess whether the type of PPE could be distinguished based on participants’ physiological data, including heart rate and activity level. This approach facilitated the analysis of the potential impact of different PPE types on user stress levels. A comprehensive flowchart detailing the entire experimental process, from data collection to analysis, is presented in Figure 1.

### 2.1. Simulation Information

The study comprised multiple simulations: 0: No activity, 1: Donning (the process of putting on PPE), 2: Listening to the lungs and heart of the patient, 3: Procedure accomplishment (e.g., starting an IV), 4: Reading urine volume from a collection bag, 5: Proning the patient (turning them face down), 6: Doffing (the process of removing PPE), 7: Status and vital signs, 8: Modifying ventilator settings. Simulations consisted of one nurse and one physician. Notably, during simulation 3, only the nurse participated. Each simulation consisted of two runs: one utilizing the Powered Air-Purifying Respirator (PAPR) and the other employing an N95 and faceshield. Consequently, a total of 22 runs of data were collected.

During the simulations, two observers were assigned to record data regarding the start and end times of each activity performed by the participants. Additionally, heart rate (HR) and movement data (steps) were captured using wearable devices, collected one day prior to the simulation as well as throughout the duration of the simulation.

This investigation was designed as an interventional randomized crossover trial, which included two distinct phases: Phase 1 involved a simulation study with ten participants, while Phase 2 consisted of field testing involving thirty participants. Both phases were conducted within a simulation laboratory and a tertiary-care hospital setting to assess the differences in performance and experiences of frontline healthcare workers during the COVID-19 pandemic [30].

**Figure 2:**
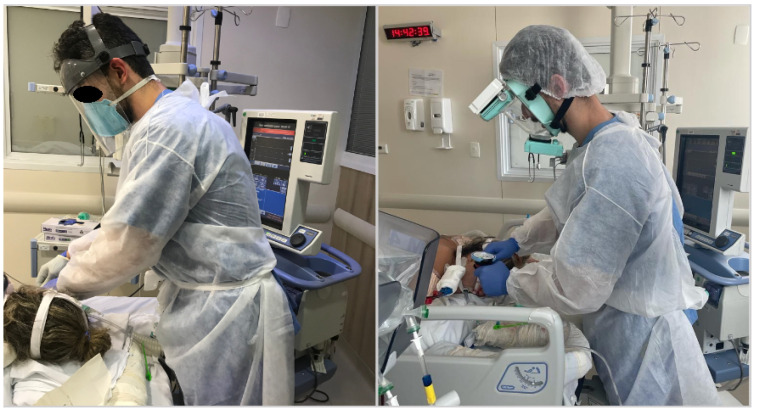
Crossover observation during field testing procedure (patient tracheal aspiration) of N95 respirator + face shield (**on the left**); and Light-Powered Air-Purifying Respirator (L-PAPR) (**on the right**). São Paulo, Brazil, 2021.

The study specifically aimed to evaluate and compare the Light-Powered Air-Purifying Respirator (L-PAPR), which integrates personal protective equipment (PPE), with a combination of a filtering facepiece (FFP2) mask and a face shield (N95/FS). The evaluation focused on several key aspects, including perceived workload, usability, incidence of usage errors, and heart rate responses. Additionally, qualitative interviews were conducted to identify barriers and enablers to the implementation of these protective measures, as well as to gather recommendations for effective training programs. Figure 2 illustrates the simulation process involving each type of PPE.

### 2.2. Data Analysis

When analyzing collected data, the goal is to find a correlation between PPE use and HR. To achieve this, the techniques Principal Component Analysis (PCA) and t-distributed Stochastic Neighbor Embedding (t-SNE) will be used. The first technique focuses on finding the main/maximum variation/correlation among the data, while the second focuses on the automatic classification of the data. In sequence, clustering validation measures will be applied to identify the quality of the formed clusters.

According to [31], PCA is a technique used to obtain the principal components, a set of orthogonal variables, from a table with several dependent variables through the extraction of major information and transformation into a new set of these variables.

The technique t-SNE is an enhancement of the SNE (Stochastic Neighbor Embedding) method. t-SNE is used to visualize high-dimensional data graphically by embedding it into a, usually, 2D or 3D space. This method converts the data into a pairwise similarities matrix and then maps these similarities to a low-dimensional space [32].

Validation of clustering results is important to ensure that the clusters formed are meaningful. In this sense, internal validation relies only on the data, without external information, which being important in cases when class labels are not available. Based on this, different measures can be used to validate a cluster internally, such as the Calinski–Harabasz index, the Davies–Bouldin index, and the Silhouette index [33].

Calinski–Harabasz is a method based on the distribution between clusters. In this score, higher values indicate better classification between the groups, and the minimum score is zero. Yet, it doesn’t have a maximum score [34].

Davies–Bouldin is calculated by taking the similarities for each pair of clusters. In this method, lower values indicate better classification between the groups. The score does not have a maximum value, and the minimum is zero [33].

The silhouette index is calculated by taking into account the mean nearest-cluster distance for each data point and the mean intra-cluster distance. The score goes from minus one to plus one. Plus one indicates that all data are correctly classified, while minus one shows that all data are best classified in the other group. Results around zero mean there is no clear way to classify the data [35].

## 3. Results

### 3.1. Data Information and Data Preprocessing

The dataset comprises a total of 1,502,730 entries. Each entry includes the following attributes:

- Datastamp: The timestamp indicating when the data point was recorded.
- Smartwatch ID: A unique identifier for the smartwatch used to collect the data.
- Heart Rate: The participant’s heart rate at the time of data collection, measured in beats per minute (BPM).
- Movement (Step): The number of steps recorded by the smartwatch.
- User: The individual participant (either a nurse or physician).
- Role: The professional role of the participant, specified as either nurse or physician.
- Simulation Run: Whether the data point was collected during the first or second run of the simulation.
- Activity ID: An identifier indicating the specific activity being performed at the time of data collection, as follows:
  - 0: No activity;
  - 1: Donning (the process of putting on PPE);
  - 2: Listening to the lungs and heart of the patient;
  - 3: Procedure accomplishment (e.g., starting an IV);
  - 4: Reading urine volume from a collection bag;
  - 5: Proning the patient (turning them face down);
  - 6: Doffing (the process of removing PPE);
  - 7: Checking patient status and vital signs at the bedside;
  - 8: Modifying ventilator settings.

The structure of the dataset allows for the examination of the relationship between heart rate, physical activity, and role-based performance during various tasks. To ensure high-quality and analyzable data, several preprocessing steps were undertaken:

1. Data Cleaning: All entries with missing or corrupted values were identified and excluded. Any inconsistencies in timestamp or smartwatch ID were corrected.
2. Time Alignment: Given that data were collected from multiple participants simultaneously, synchronization of the timestamps across devices was performed to ensure that heart rate and movement data corresponded accurately to specific activities and time periods within the simulation.

Table 1 presents an excerpt from the dataset, illustrating the variety of activities and physiological responses recorded during the simulation. This dataset, after preprocessing, provides a comprehensive basis for further analysis of the physiological and psychological impacts of using PAPR compared to the N95 respirator and face shield.

From the dataset, a total of 52,683 data entries were matched specifically from the simulation phase, which represents 14.6 h of data collection. This amounts to an average of 40 min of testing for each participant during the simulations.

During the simulation, heart rate (HR) values were recorded, ranging from 48 to 130 beats per minute (bpm). Heart rate measurements were taken every second, allowing for a detailed analysis of physiological responses throughout the simulation. The remaining 1,450,046 entries in the dataset were collected before and after the simulation events, providing a comprehensive view of participants’ heart rate and activity levels outside the simulation context.

The movement data were obtained from an accelerometer sensor embedded within the smartwatch, which quantified physical activity in terms of steps. The smartwatch’s algorithm aggregated step counts at irregular intervals, necessitating a method to standardize these measurements for subsequent data analysis. To achieve this, we calculated the average speed of movement in steps per second. While this metric may not capture precise movement dynamics, it offers valuable insight into overall user activity levels.

Additionally, the activities performed during the simulation were documented by observers, who recorded the start and end times for each activity. Each entry in the dataset was then labeled accordingly to facilitate accurate analysis.

**Figure 3:**
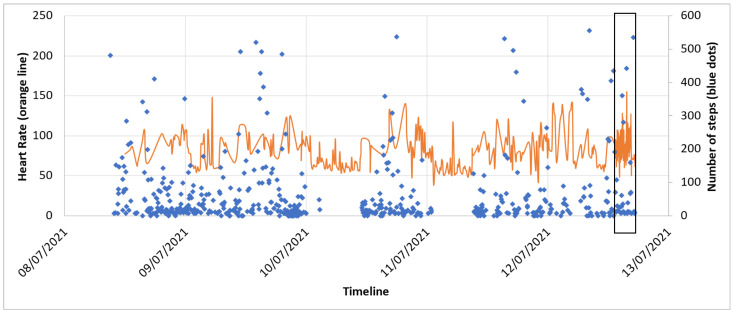
Heart rate and movement over time for participant 7. The black box delineates the duration of the observations made during the simulation. São Paulo, Brazil, 2021.

Figure 3 illustrates the collected data for one specific user (participant 7). The heart rate measurements are represented by an orange line graph on the left scale, while the number of steps is plotted using blue diamonds on the right scale, which employs a secondary axis. Black boxes on the graph delineate the duration of the observations made during the simulation, providing a visual context for the data collected.

### 3.2. Analysis Results

The initial analysis aimed to determine whether we could distinguish between the use of the N95 respirator with a face shield and the Powered Air-Purifying Respirator (PAPR) based solely on heart rate (HR), movement data, participant characteristics, and activity identifiers. To achieve this objective, we employed two advanced statistical techniques: Principal Component Analysis (PCA) and t-Distributed Stochastic Neighbor Embedding (t-SNE).

Principal Component Analysis (PCA) was utilized to reduce the dimensionality of the dataset while retaining the variance that is most informative. By transforming the original variables into a new set of uncorrelated variables (principal components), PCA helps in identifying patterns and relationships in the data that may not be immediately apparent. All graphs using PCA show an array of parallel lines. This visualization is in accordance with the heart rate data, which is rounded to integer numbers. As PCA keeps the data linearity intact, this artifact is expected.

Following PCA, we applied t-SNE, a technique designed for visualizing high-dimensional data by embedding it into a lower-dimensional space (typically 2D or 3D). t-SNE focuses on preserving the pairwise similarities between data points, allowing us to cluster similar observations while minimizing the distance between points in the same cluster.

**Figure 4:**
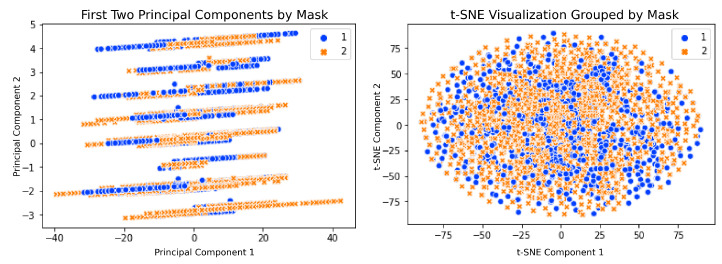
Visualization of heart rate and movement data using Principal Component Analysis (**left**) and t-SNE (**right**). ‘1’ (blue circles) represents the N95 respirator with a face shield, and ‘2’ (orange crosses) represents the PAPR. Visual inspection reveals a high degree of data overlap, demonstrating no distinct or separable clusters for either PPE type.

The graphical results of this analysis are presented in Figure 4. This figure illustrates how participants’ HR and movement data cluster according to the type of respiratory protection used, thereby enabling a visual differentiation between the N95 respirator with a face shield and PAPR. The clustering patterns observed in the figure suggest no potential differences in physiological responses and movement behaviors associated with the two types of PPE during the simulation.

**Figure 5:**
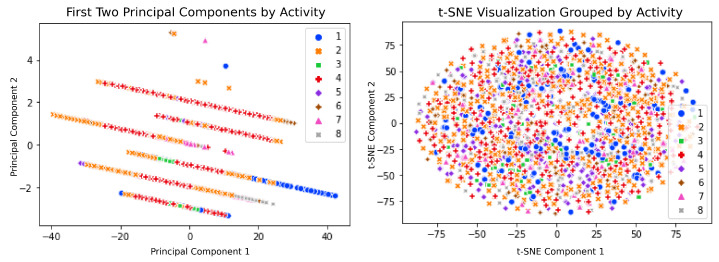
Visualization of heart rate and movement data using Principal Component Analysis (**left**) and t-SNE (**right**), classified by the specific activity performed. The numbers 1 through 8 in the legends correspond to the distinct simulation tasks, as detailed in Section 3.1. Visual inspection reveals a high degree of data overlap, demonstrating no distinct or separable clusters associated with varying levels of task difficulty.

**Figure 6:**
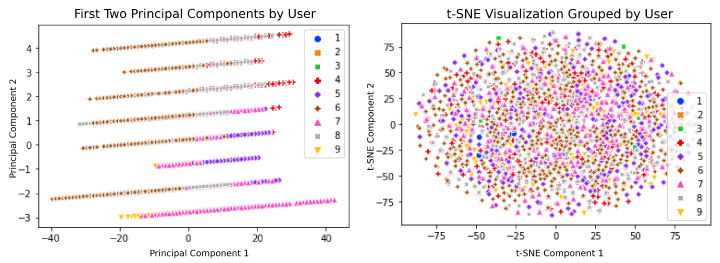
Visualization of heart rate and movement data using Principal Component Analysis (**left**) and t-SNE (**right**), classified by individual participant. The numbers 1 through 9 in the legends correspond to the users in the simulation. Visual inspection reveals a high degree of data overlap, demonstrating no distinct or separable clusters associated with individual physiological baselines.

Given the lack of visual separation in Figure 4 we delved deeper into the data by conducting additional analyses aimed at identifying potential differences based on specific activities and individual users. The outcomes of these analyses are presented in Figure 5 and Figure 6, respectively.

Analysis by User: In this analysis, we sought to uncover the underlying differences in each participant’s physiological response—referred to as their “internal biomarker.” By examining HR and movement data on a per-user basis, we aimed to identify how individual characteristics may influence physiological metrics during the use of different PPE. This analysis allows for a more personalized understanding of how different healthcare workers respond to the same environmental stress.

Analysis by Activity: In parallel, we evaluated the data based on the activity performed during the simulations. This was done to show the differences between calm/easy activities and demanding/hard activities. This approach aimed to highlight the differences in HR and movement data associated with varying levels of task difficulty. By doing so, we can better understand how the intensity of activities influences physiological responses, potentially offering insights into the workload and stress experienced by healthcare providers during specific procedures.

However, visual inspections across all these classifications (PPE, user, and activity) revealed no clear, separable clusters. To objectively quantify this and provide a more robust evaluation, we utilized three metrics of cluster separation (Table 2): the Calinski–Harabasz index, the Davies–Bouldin index, and the Silhouette score.

As indicated in Table 2, the Calinski–Harabasz scores are high only for users on PCA, suggesting a poor classification of the clusters in the other analyses. Low scores in the Calinski–Harabasz index imply that the variance between clusters is not significantly greater than the variance within clusters, indicating a lack of clear separation.

Conversely, the elevated Davies–Bouldin scores (ranging from 4.58 to 60.91) clearly indicate poor clustering and reflect a high similarity among the groups. Even the best value (4.58 for the user on PCA analysis) demonstrates that we cannot adequately separate the clusters. This suggests that the data analysis, whether employing Principal Component Analysis (PCA) or t-Distributed Stochastic Neighbor Embedding (t-SNE), is unable to reliably distinguish between clusters. With this result, the high value for the user variable found in PCA analysis by the Calinski–Harabasz index cannot be considered indicative of meaningful clustering.

To strengthen the analysis and confirm the results obtained by the Davies–Bouldin index, the Silhouette score was utilized to provide further clarity regarding the clusters. With values near zero across all classifications, these results demonstrate a high degree of overlap, indicating that we are unable to reliably differentiate between the N95+Face shield and the PAPR using the available heart rate and movement data.

Consequently, rather than definitively proving identical stress responses, these findings highlight the methodological limitations of the clustering methods in identifying meaningful differences in heart rate and movement data associated with the use of Powered Air-Purifying Respirators (PAPRs) compared to N95 respirators with a face shield during the simulations.

## 4. Discussion

### 4.1. Practical Implications for PPE Policy

Although the heart rate data did not reveal significant differences between the two types of personal protective equipment, these findings offer practical relevance for healthcare policy. Existing literature indicates that L-PAPRs provide distinct operational and safety advantages over N95 respirators combined with face shields, including enhanced protection via continuous positive pressure, better visualization of facial expressions, and the elimination of heat accumulation [30]. While L-PAPRs may introduce specific communication difficulties due to airflow noise [30], our study found no elevated gross physiological burden associated with their use. Consequently, within the limitations of this study, no physiological drawbacks were observed that would offset the established benefits of L-PAPR use.

### 4.2. Limitations and Future Directions

While this exploratory study provides initial insights into the heart rate and movement profiles associated with different PPE, several methodological limitations must be explicitly acknowledged:

- *Environmental Generalizability:* These findings may not universally apply across all clinical settings. Variations in environmental factors (e.g., temperature, humidity) and operational demands (e.g., shift lengths, department types) can significantly alter the physiological burden of PPE, requiring caution when extrapolating these results.
- *Biomarker Sensitivity and Psychological Validation:* The primary limitation is the reliability on heart rate (HR) rather than heart rate variability (HRV), which is a definitively more sensitive indicator of cognitive load. Furthermore, the absence of validated psychological ground truth, such as cortisol measurements or standardized Perceived Stress Scale surveys, restricts the ability to isolate psychological stress from general physical effort.
- *Device Accuracy and Noise:* Data was collected using consumer-grade Withings Steel HR smartwatches. While useful for continuous field monitoring, these devices lack the clinical-grade accuracy of reference devices (e.g., standard ECG chest straps), introducing potential measurement noise and artifact interference into the dataset.
- *Sample Size and Statistical Power:* The study was conducted with a relatively small group (10 participants in simulation, 30 in the field) without an a priori power analysis. This limited sample size introduces a risk of Type II error (false negatives), meaning the study may simply have lacked the statistical power required to detect subtle, yet real, differences in physiological responses between the PPE types.
- *Analytical Constraints and Confounding Factors:* The analysis relied heavily on unsupervised machine learning methods (PCA and t-SNE) without labeled stress data. Additionally, the study lacked a baseline normalization strategy to account for confounding factors, including individual baseline fitness levels, the specific metabolic demands of different tasks, and circadian variations throughout the shift.

While the analyzed dataset reflects observations from a specific operational period, establishing this exploratory baseline serves to inform and justify the design of new experiments. To address the combined limitations identified above, future investigations must transition from this foundational pattern recognition to formal hypothesis testing. Specifically, subsequent studies should employ appropriate inferential statistical methods to rigorously quantify the physiological impacts of different PPE, allowing for robust statistical comparisons while formally controlling for the confounding variables addressed above.

## 5. Conclusions

In accordance with existing literature, heart rate variability (HRV) is recognized as a viable indicator of stress levels and cognitive load. However, our analyses—specifically Principal Component Analysis (PCA), t-Distributed Stochastic Neighbor Embedding (t-SNE), and various clustering metrics—reveal that HR alone cannot effectively differentiate between the types of PPE used (PAPR versus N95 respirator and face shield), regardless of whether individual participants or specific activities are examined. Furthermore, we found no significant differentiation in HR based on either user or activity, even when including movement information in our analysis.

It is important to note that our study was limited to HR measurements due to the unavailability of heart rate variability (HRV) data, which is often a more sensitive indicator of stress and cognitive load. Given this limitation, no detectable differences were observed using HR-based analysis under the current experimental conditions. The inability to detect differences in stress or cognitive load may stem from the limitation of HR as a biomarker, rather than an actual absence of stress variations. Consequently, we conclude that neither PPE demonstrates inferiority regarding heart rate impact, but further investigations utilizing HRV are required.

Further research in this area is warranted, particularly studies that incorporate heart rate variability (HRV) measurements to provide a more nuanced understanding of how different PPE affect users’ physiological responses, especially given HRV training’s promise for stress reduction. Moreover, investigating additional physiological and psychological markers could offer a more comprehensive perspective on how various PPE types and activities impact healthcare workers in high-stress environments. These expanded studies would contribute valuable insights to inform evidence-based practices for optimizing healthcare worker well-being and performance.

## Figures and Tables

**Table 1:** Samples from the dataset.

Entry ID	Time Stamp	Watch ID	HR	Steps/s	Part. #	Role	Mask	Sim Run	Activity
1210	19 July 2021 09:32:15	1	96.0	0.00	9	0	1	9	1
1211	19 July 2021 09:32:17	1	94.0	0.00	9	0	1	9	1
1212	19 July 2021 09:32:20	1	94.0	0.00	9	0	1	9	1
1213	19 July 2021 09:32:21	1	94.0	0.00	9	0	1	9	1
1214	19 July 2021 09:32:22	1	94.0	0.00	9	0	1	9	1
⋮	⋮	⋮	⋮	⋮	⋮	⋮	⋮	⋮	⋮
1418004	12 July 2021 16:21:56	10	110.0	0.15	6	1	1	8	4
1418005	12 July 2021 16:21:57	10	109.0	0.15	6	1	1	8	4
1418006	12 July 2021 16:21:57	10	109.0	0.15	6	1	1	8	4
1418007	12 July 2021 16:21:58	10	109.0	0.15	6	1	1	8	4
1418008	12 July 2021 16:21:58	10	109.0	0.15	6	1	1	8	4

**Table 2:** Results of clustering measures.

	Calinski–Harabasz		Davies–Bouldin		Silhouette Score	
	(Higher Is Better)		(Lower Is Better)		(0 Is Worse)	
Variable	PCA	TSNE	PCA	TSNE	PCA	TSNE
Mask	439.70	147.78	9.06	17.08	0.014	0.004
Act	509.72	42.09	52.35	60.91	−0.206	−0.078
User	7098.45	40.39	4.58	35.05	−0.335	−0.330

## Data Availability

Given the sensitive nature of this data and our data management and IRB agreements in place to protect participants, data can be made available only on a case-by-case basis. If you require access to this data please contact the corresponding author for the next steps.

## Abbreviations

The following abbreviations are used in this manuscript:

BPM	Beats Per Minute
EDA	Electrodermal Activity
HCWs	Healthcare Workers
HR	Heart Rate
HRV	Heart Rate Variability
L-PAPR	Light-Powered Air-Purifying Respirator
LSTM	Long Short-Term Memory
PAPR	Powered Air-Purifying Respirator
PCA	Principal Component Analysis

PPE	Personal Protective Equipment
SVM	Support Vector Machine
t-SNE	t-Distributed Stochastic Neighbor Embedding
WHO	World Health Organization
